# Pattern segmentation with activity dependent natural frequency shift and sub-threshold resonance

**DOI:** 10.1038/srep08851

**Published:** 2015-03-09

**Authors:** E. Shtrahman, M. Zochowski

**Affiliations:** 1 Applied Physics Program, University of Michigan – Ann Arbor 48109, USA; 2 Department of Physics, University of Michigan - Ann Arbor 48109, USA; 3 Biophysics Program, University of Michigan - Ann Arbor 48109, USA

## Abstract

Understanding the mechanisms underlying distributed pattern formation in brain networks and its content driven dynamical segmentation is an area of intense study. We investigate a theoretical mechanism for selective activation of diverse neural populations that is based on dynamically shifting cellular resonances in functionally or structurally coupled networks. We specifically show that sub-threshold neuronal depolarization from synaptic coupling or external input can shift neurons into and out of resonance with specific bands of existing extracellular oscillations, and this can act as a dynamic readout mechanism during information storage and retrieval. We find that this mechanism is robust and suggest it as a general coding strategy that can be applied to any network with oscillatory nodes.

The brain forms a complex interconnected network in which spatio-temporal patterns of neuronal activity are thought to underlie its information processing. However, basic questions remain unanswered. What aspects of this patterning represent the information content? How do these network wide representations robustly emerge out of the dynamical properties of individual neurons? How are distributed features dynamically bound together, while at the same time segmented out from the rest of the network, to form a coherent representation? These questions not only pertain to the brain, but also to the formation of distributed representations in any network. In terms of neuronal processing, many proposed coding schemes are based on either the firing rate or the temporal structure of spikes in response to a stimulus^1^,^2^,^3^. Correlations within these activity patterns lead to network rewiring, where the connections between neurons encoding the same functional pattern are strengthened. However, it is unclear how these regions of enhanced connectivity or biased input can lead to virtually instantaneous, dynamically evolving, and robust separation of activity patterns, which in turn encode for functionally diverse information content, while embedded within the same interconnected group of neurons.

Single neurons integrate input to generate action potentials, however they can also display damped sub-threshold oscillations giving them complex dynamics and sensitivity to temporal patterns of input. The ability to resonate at particular frequencies has been observed in many experimental preparations^4^,^5^. The natural frequency has been shown to be voltage dependent and it can shift at both depolarized and hyperpolarized membrane potentials^6^,^7^,^8^. This phenomenon was reported in various cell populations such as hippocampal pyramidal neurons^6^,^7^ and pyramidal cells of the amygdala olfactory cortex^8^. In the case of hippocampal pyramidal cells, the underlying ionic mechanisms are related to a slow hyperpolarization-activated cation current (often referred to as I_h_ current), a slow activating potassium current (so called M-current as it acts through muscarinic receptors), and an instantaneously activating, inwardly rectifying potassium current. Interestingly, these mechanisms are activated differentially when a cell is hyperpolarized (I_h_ is activated) or depolarized (I_M_ is activated). In all cases, however, voltage gated sodium channels played a central role, as application of tetrodotoxin (TTX) abolished the sub-threshold oscillations^6^,^8^. The observed resonance shifts range from a couple of Hz to more then 10 Hz.

At the same time oscillations of large neuronal populations have been observed with local field potentials (LFP) or EEG measurements^9^,^10^,^11^, and these oscillations are classified into discrete frequency bands spanning single to tens of Hertz. These brain rhythms have been implicated in various cognitive functions^12^,^13^. However, the influence of these oscillations on individual neuronal activity patterns or network wide activity has remained unclear^14^. Sub-threshold oscillatory input through synaptic or ephaptic coupling provides feedback between brain oscillations in the local field potential and individual neurons^15^. This idea is experimentally supported by findings that oscillations in the visual system can affect downstream processing, as seen in the phase locking of neurons in the LGN to the 50 Hz oscillations in the input received from the retina^16^.

We propose a novel mechanism that links the voltage-dependent resonance frequency shifts of individual neurons with the large scale oscillatory rhythms observed in the brain to selectively activate neuronal ensembles. We hypothesize that sub-threshold depolarization from synaptic coupling or external input can shift neurons into and out of resonance with specific bands of extracellular oscillations and this resonance shift can act as a mechanism to selectively activate functionally diverse neural populations. Thus a given oscillatory band acts as a readout carrier for neurons that are selectively depolarized and shifts their resonance frequency into that band. We investigate this mechanism for memory storage and retrieval but it could also pertain to rapid attention switching^17^,^18^,^19^. We show that this is a robust mechanism that works within broadly defined known biological constraints^14^,^20^ and can explain experimentally observed neuronal dynamics during the storage and retrieval processes^21^,^22^,^23^. It also provides a mechanism for dynamic signal separation within a network that can change rapidly as a function of external input and network structure, or both. It provides flexibility and easily combines patterns of external stimuli with intrinsic structure for emergent readout of activity patterns. The proposed mechanism is not limited to neuronal networks but it is a universal dynamical mechanism that can be more generally applied to other networks. As we show below, the only two required features are that the network is composed of oscillatory nodes whose natural frequencies vary as a function of the input magnitude and a readout frequency.

## Results

To explore this pattern separation mechanism in the brain context, we investigated maximally reduced neuronal networks fitting the above criteria. Namely, we constructed networks composed of 400 Resonate-and-Fire neurons^24^. These neurons are essentially damped oscillators that emit an action potential when the voltage reaches a threshold. In addition, the neurons have a current dependent resonance frequency and are driven by a weak (sub-threshold) external oscillatory current (see Methods).

**Figure 1:**
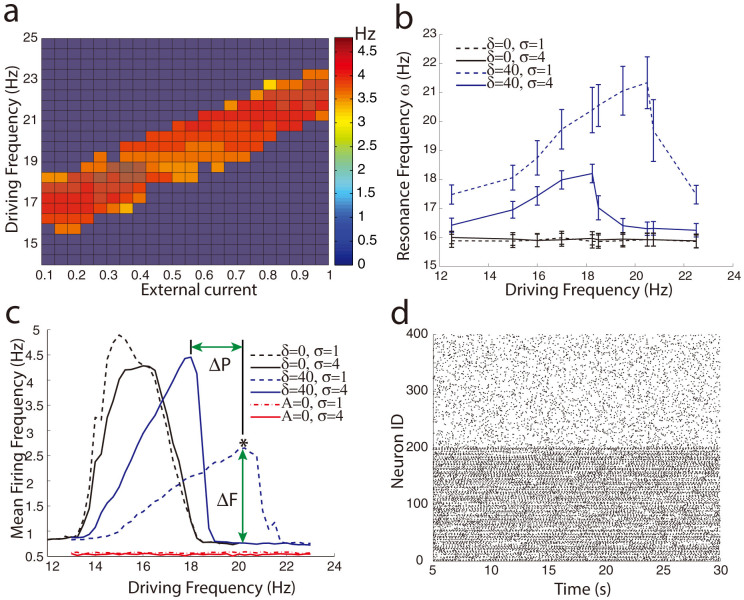
Intrinsic resonance frequency shifts is based on current input and allows for selective activation of regions of a network. (a) Single RF neuron firing frequency response to varying additional external current (I_sub_) and driving frequency of sub-threshold current input. (b) The average resonance frequency ω in a network with (blue) and without (black) resonance frequency shift (δ = 40 and δ = 0, respectively). A sub-network (dashed lines) with additional coupling from synaptic multiplier (σ = 4), has a shifted average resonance frequency from neurons outside of the heterogeneity (solid line). (c) Resonance curves of network firing across driving frequencies, shows a shift in resonance for the network heterogeneity (σ = 4). For non-oscillatory current input (A = 0), resonance is abolished. (d) Example raster plot of network activity for sub-threshold driving frequency of 20 Hz. A subset of the network, Neurons 1–200, have an enhanced coupling multiplier of σ = 4 and are selectively activated by the sub-threshold oscillatory input.

We impose the experimentally observed voltage dependent resonance frequency shift^6^,^7^,^8^ by Eq. (2), (see methods) and observe the resonance phenomena as we vary the driving frequency of the sub-threshold oscillatory input current (Fig. 1). The firing frequency of neurons preferentially increases when the driving frequency matches the resonance frequency of the neuron. The cells shift their resonance in response to the applied current input (Fig. 1a). In a network, this additional current input to a single neuron can arise from co-activation of the neurons having higher synaptic coupling. We refer to regions of the network containing additional synaptic connectivity as network heterogeneities. Neurons within network heterogeneities receive additional sub-threshold current input from their synaptic connections. This current shifts the resonance frequencies of neurons within the heterogeneity to higher frequencies (Fig. 1b), separating the response frequencies of the heterogeneity from rest of the network (Fig. 1c), and thus allowing for selective activation of the heterogeneity for matching driving frequencies (Fig. 1d). Thus, even though all neurons in the network are driven with the same sub-threshold oscillatory input current representing ongoing brain rhythms, the resonance frequency shift allows higher connectivity regions to separate out from the rest of network in their firing rate profiles.

Figure 1c depicts the network wide resonance curves (i.e. mean firing frequency within the given neural population) when there is no cellular resonance frequency shift (δ = 0) and with current dependent resonance frequency shift (δ = 40) for two different coupling strengths (coupling multipliers σ = 1 and σ = 4, please refer to methods section below). We observe a significant shift of the network response towards higher driving frequencies with increased coupling strength, when the neuronal resonance frequency is current-dependent. At the same time the shape of the resonance curves changes significantly, and can be understood by considering the distribution of resonance frequencies in the network. The response of the network region with lower coupling is narrower but taller indicating, on one hand, higher response specificity to the frequency of the driving oscillation, and at the same time an increased network response magnitude in terms of an elevated mean network firing frequency. This is due to the fact the variance in the resonance frequencies of the network of neurons increases significantly with network coupling strength (error bars on Fig. 1b) because of the higher variability in the total input the neurons receive. This in turn causes some neurons to be in resonance with the driving current while others remain out of resonance, which makes the network response as a whole change gradually and achieve lower overall values. The network achieves maximal response when the driving frequency matches the resonance frequency of most neurons in the network. This variation in the resonance frequencies of neurons in the network also explains the asymmetry in the resonance peak. When the driving frequency is high there are fewer neurons that resonate with the driving oscillatory current (note the widest distribution of resonance frequencies is at the falling phase of the network resonance curve, Fig. 1b). Their number quickly increases as the driving frequency is lowered. For intermediate values of driving frequency there is a finite pool of neurons that resonate and activate the network-wide response.

This separation mechanism is robust across a wide range of parameters. We quantified the separation mechanism with two metrics, the peak-to-peak distance ΔP and the resonance firing frequency difference ΔF (please refer to Figure 1c). We explored the separation of network activation for various network connectivity strengths as a function of the network topology, strength and frequency of external oscillatory current, noise level and magnitude of activity dependent resonance shift. The network topology is varied using the small world network paradigm^25^, with additional network heterogeneities added in the coupling weight within neuronal subgroups of the network.

**Figure 2:**
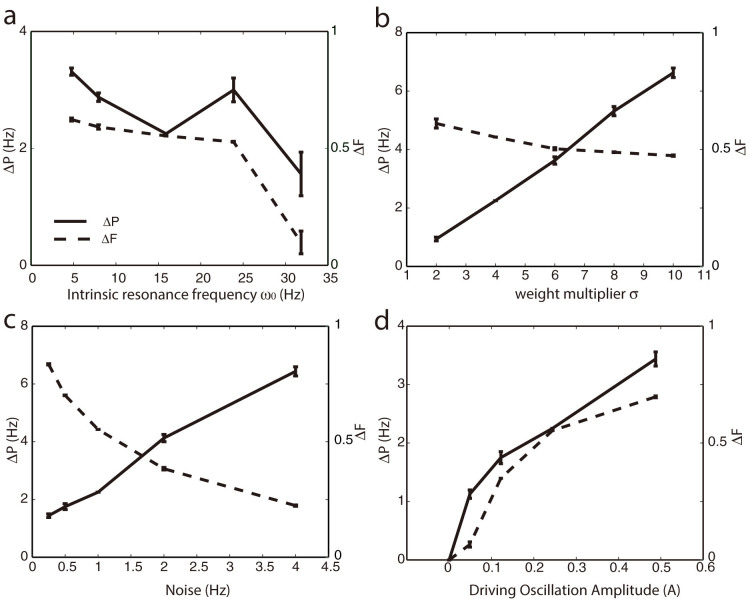
Robustness of the resonance based separation mechanism as a function of various system parameters for disconnected networks. Separation of signals is characterized with peak to peak distance ΔP (solid lines) and normalized firing frequency difference ΔF (dashed lines) for changing intrinsic cellular resonance frequencies ω_o_ (a), additional coupling weight σ (b), noise (c), and amplitude of sub-threshold oscillatory current A (d). For both ΔP and ΔF values above zero denote significant separation.

To better understand the workings of the proposed mechanism, we quantify the signal separation, both ΔP and ΔF, for various system parameters (Fig. 2). To investigate the sole effect of synaptic strength on signal separation for different simulation parameters, we eliminated the connections between the heterogeneity and the rest of the network, which we refer to as disconnected networks. For both metrics (ΔP and ΔF), values above zero denote significant separation; all error bars represent 1 standard error of the mean, and all means are averaged over 4 simulations. As expected, the peak-to-peak separation increases with larger values of the resonance frequency shift δ (not shown). Both measures report separations of several Hz for varying values of δ. Results presented in Figure 2 are for disconnected networks, therefore the effects on separation are due to disparate connectivity strengths alone. The separation mechanism is not limited to particular resonance frequencies of the neurons. However, the measures report weaker separation at higher resonance frequencies (Fig. 2a). The peak-to-peak distance increases while the resonance firing frequency difference decreases (Fig. 2b, c) for higher coupling weight within the network heterogeneity or higher driving noise. The first is due to the fact that both of these quantities provide additional activity levels (or current) to recipient neurons increasingly shifting their resonant frequency away from the baseline. However since neurons can have a variable number of inputs, stronger coupling increases the spread of instantaneous resonance frequencies, which widens but reduces the height of the resonance peak. We have also investigated the size of the external oscillatory current required to observe signal separation. We report its magnitude (Fig. 2d) as a ratio to a minimal constant threshold current needed to generate a spike. Robust signal separation appears for oscillatory amplitude as low as 0.1 of the threshold current, well within those observed experimentally^15^,^26^ (Fig. 2d).

**Figure 3:**
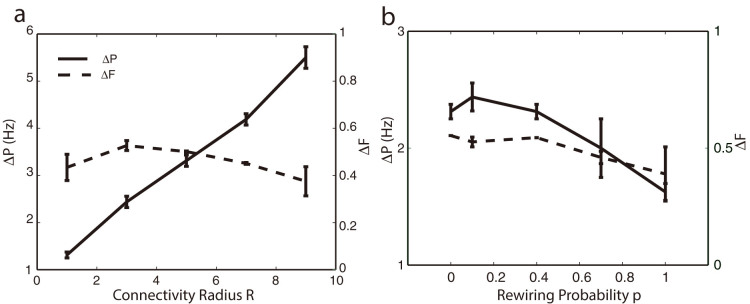
The influence of network topology on separation measures for connected network clusters. (a) Peak to peak separation ΔP (solid lines) increases for larger connectivity radius R. (B) Signal separation as a function of network topology (rewiring parameter p).

We then investigated how the signal separation changes for two interconnected regions as a function of the network topology and the number of connections (Fig. 3). We vary the network topology from local to random coupling by changing the connection rewiring probability^25^. We find that this separation mechanism is effective across various network connectivity parameters. Both separation measures are only marginally influenced by the rewiring probability, showing a small decrease of separation for increasingly random networks. This is due to the fact that for more random networks there are relatively fewer connections within the functional subgroups while the subgroups are more tightly interconnected. At the same time, peak-to-peak separation increases for a higher radius of connectivity, as it provides additional input variance between the neurons within the heterogeneity and outside of it.

**Figure 4:**
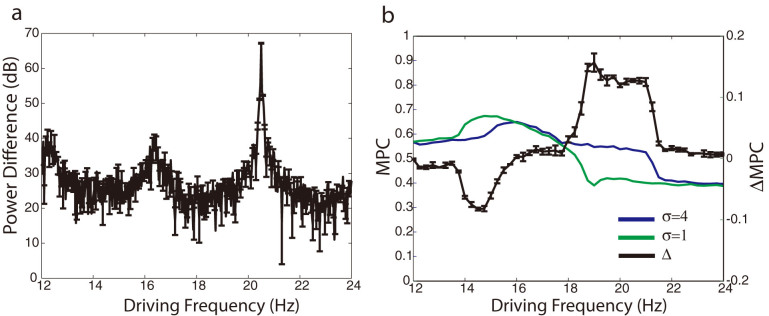
Total activity within heterogeneity shows separation in frequency content and increased phase coherence with the sub-threshold driving oscillation when in resonance. (a) Difference in power of total network activity (heterogeneity – outside heterogeneity) shows a peak in the power spectrum for resonant frequency of driving oscillation (marked with * in fig. 1). (b) Difference in Mean Phase Coherence between sub-threshold oscillatory input and the activity signal formed within (blue) and outside (green) the heterogeneity.

The above results indicate that we see a robust separation in the frequency response of the neurons forming the network heterogeneity from the rest of the network. It has been shown that hippocampal memory formation both in animals and humans is accompanied by increased power in the theta band oscillation as well as phase coherence of neuronal activity with oscillations at that frequency^21^,^22^,^23^. We hypothesize that the experimentally observed increase in power, for example of theta during memory consolidation, as well as the increased phase coherence of neuronal activities with that band, can be explained by our resonance readout mechanism, as it is well established that the resonating oscillators lock to driving oscillatory signals^27^. To that effect we investigated the difference in the power spectrum of the average activity within and outside of network heterogeneities. We compared the power spectrum of the average activity within the network heterogeneity to the rest of the network and found a notable increase in power when the driving oscillation is in resonance with the network heterogeneity (Fig. 4a). To better understand the increase of power around the driving frequency, we measured changes in the phase locking between the neurons within and outside of the heterogeneity as a function of driving frequency. To quantify the phase locking we computed the instantaneous phase of the network activity. Using the phase difference between the network activity and the sub-threshold driving oscillation, we calculated the Mean Phase Coherence (MPC)^28^, and found a substantial increase in mean phase coherence between the driving oscillation and the heterogeneity at its resonant peak (Fig. 4b). Here we depicted the mean phase coherence as a function of the driving frequency for the heterogeneity (blue line), for the rest of the network (green line), and for the difference between the two (black line). We observe that the mean phase coherence between the network firing and the driving oscillation is relatively highest at the raising phase of the network resonance curve peaks for both the heterogeneity and the rest of the network. Thus the proposed resonance frequency shift mechanism separates out the cell activities within a network heterogeneity for a significant range of driving frequencies in both their firing rate and phase coherence with ongoing oscillations. It is interesting to note that the mean phase coherence drops rapidly on the falling phase of the resonance curve. This again can be attributed to the large heterogeneity in cellular resonance frequencies, with relatively few neurons driving the network-wide response.

**Figure 5:**
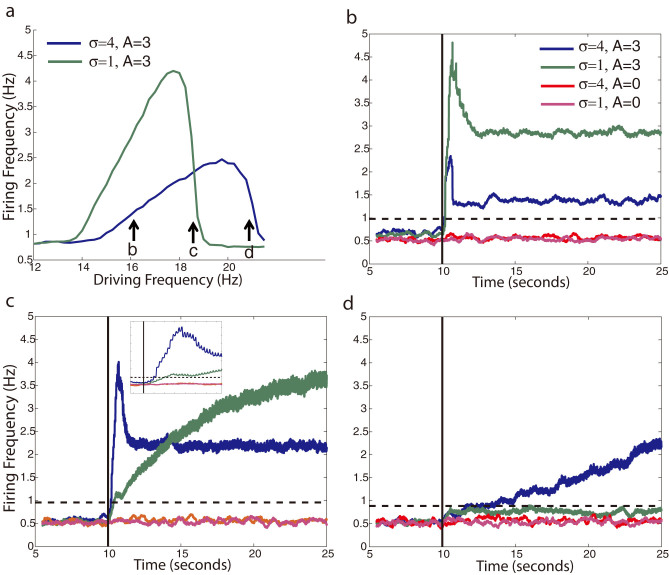
Response time to current induced resonance shift. (a) Example resonance curves for a network after additional current input begins 10 seconds into the simulation. The current input shifts the resonance frequency of the neurons as marked by the arrows. (b–d) response times of the network depicted on (a) 16 Hz (b), 18.5 Hz (c), and 21 Hz (d). Solid black line marks the onset of additional sub-threshold current. The average instantaneous firing rate over time is plotted for neurons within and outside of the network heterogeneity (σ = 4 and σ = 1, respectively) and also for non-oscillatory current input (A = 0). The inset in (c) shows the magnified timescale of firing onset. Black dashed lines mark 5 standard deviations above the baseline firing rate. All firing rate curves are averaged over 4 simulations.

We have shown that current dependent resonance shifts can separate out regions of a network with higher coupling in both their firing frequency and phase locking to sub-threshold oscillatory input. We then investigated the response time of the network to new patterns of input in order to understand how long the network takes to form separated representations in response to shifting resonances. Here the timescale is set by the frequency of the oscillatory input and can potentially be directly compared with experimental findings measuring response time to the incoming stimulus^29^,^30^,^31^. We investigated the timescale of pattern formation by observing the response time required to reach the significantly elevated firing rates observed in the resonance curves. Specifically, to probe this onset time of enhanced resonant firing, we applied an additional sub-threshold current input during the simulation and measured the time delay between this input and the significant change (see methods) in neuronal firing rate (solid black lines in Fig. 5). As before, we simulated the sub-networks having two strengths of coupling constants σ. We found that the response time critically depends on the relative position (in frequency space) of driving frequency with respect to network resonant curve (Fig. 5a). The network response time to the external input on the rising portion of the resonance curve (Fig. 5b) is significantly faster than on the falling portions (Fig. 5c,d). The shifted resonance curve of the heterogeneity (high σ) relative to the rest of the network (low σ) results in the rise and decline portions of their resonance curves to occur for different driving frequencies. Therefore, we observe that for driving frequencies where the resonance curves overlap (Fig. 5c) and the neurons in the heterogeneity are on the upward sloping portion of their resonance and the neurons outside of the heterogeneity are on the downward portion, the response times are widely different. (Fig. 5c inset).

This variation in the timescale for response times can be explained by the behavior of the network's mean phase coherence at different driving frequencies, and by the resonance frequency distribution. When the mean phase coherence is large and a significant number of neurons are active (i.e. they are in resonance), even a small perturbation in the current input drives a rapid network response, as the active neurons synchronously respond to perturbation. Here the response happens within a single cycle of the driving oscillation. However, if the neurons are largely desynchronized the rapid response to the current perturbation is not observed and the slow drift in the network response can be attributed to slow changes in the average cellular resonance frequencies due to the current input. This large variation in the response times potentially provides an additional mechanism of signal separation.

**Figure 6:**
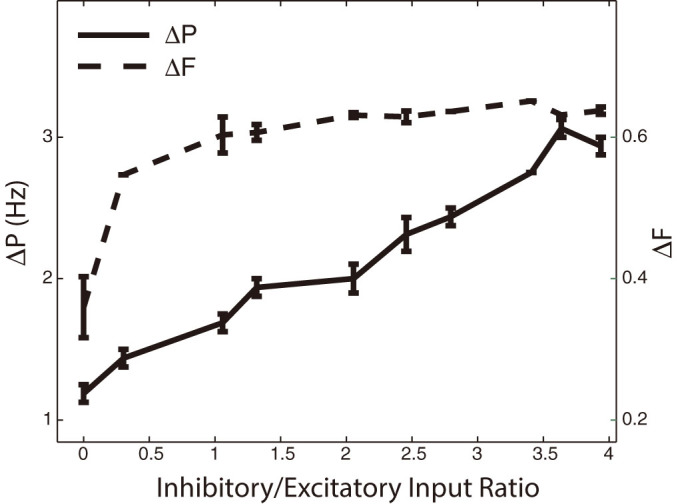
Firing rate difference ΔF (dashed line) and the peak-to-peak distance ΔP (solid line) as a function of inhibitory coupling strength. Inhibitory network strength is shown as a ratio of the inhibitory synaptic input to excitatory synaptic input (see methods). The separation mechanism is enhanced for networks in the balanced state (Inhib/Excit input ratio = 1), and also for larger inhibitory to excitatory input ratios, even with weaker resonance frequency shift (results shown are for δ = 10). Values for Inhib/Excit input ratio are given for the peak of the resonance curve (18 Hz driving frequency).

We have shown a potential mechanism for ensemble activation in simplified excitatory only networks. However, inhibition can potentially play an important role in further separating the activity of the functionally disparate network regions. To illustrate this we formed and coupled an additional network of 400 inhibitory Integrate-and-Fire^32^ neurons to the existing excitatory network such that the activity of the heterogeneity and rest of the network are mutually inhibitory. We varied the strength of the inhibitory coupling and found that inhibition enhances the separation between the activated patterns (Fig. 6). This effect is not surprising as the inhibitory neurons are activated by the corresponding resonating excitatory population and inhibit other excitatory neurons, driving their resonance frequencies away from the driving frequency at the same time. To quantify the strength of inhibition in relation to the strength of excitation, we show the separation between activated patterns as a function of the ratio of the mean total inhibitory to excitatory inputs that the cells receive over time. The ratio of one corresponds to a balanced network (Fig. 6).

**Figure 7:**
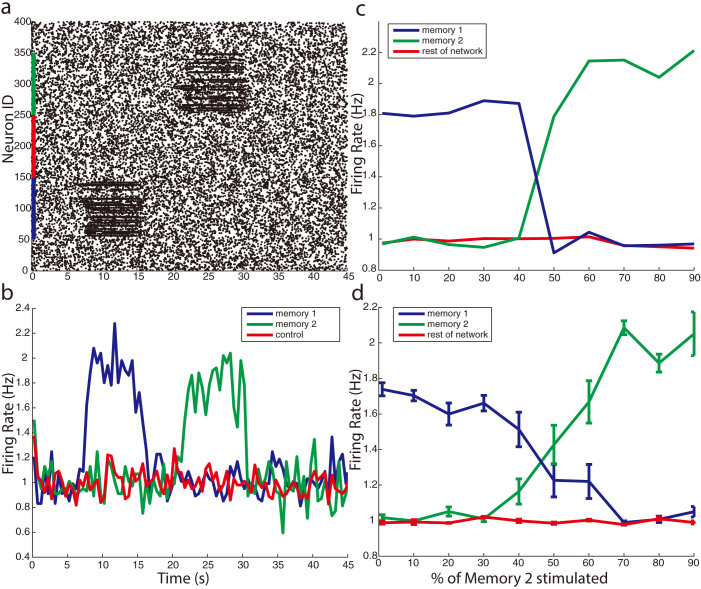
Resonance Frequency shift mechanism can selectively activate multiple competing heterogeneities through lateral inhibition. (a) Example raster plot shows selective switching between heterogeneities for a driving frequency of 25.5 Hz. Neurons with IDs 50 to 150 belong to one heterogeneity, while neurons with IDs 250 to 350 are in a second heterogeneity. Between 5 and 15 seconds of the simulation heterogeneity 1 is shifted into resonance with sub-threshold input applied a randomly chosen subset of 50% of neurons in that heterogeneity. Similarly, Heterogeneity 2 is shifted into resonance with sub-threshold input, between 20 and 30 seconds of the simulation. As a control, a region outside of both heterogeneities (IDs 150 to 250) receives identical sub-threshold input between 35 and 45 seconds in the simulation, and results in no enhanced firing rate. (b) Corresponding firing rate responses of regions of the network to sub-threshold input shows multiple heterogeneities can be selectively activated. (c) Targeted inhibition can dynamically and rapidly switch activation between heterogeneities. Heterogeneity 1 is activated by sub-threshold stimulation of 50% of neurons belonging to that heterogeneity; the x-axis denotes the percentage of stimulated cells in second heterogeneity. The activation of heterogeneities switches rapidly as number of stimulated neurons in heterogeneity 2 grows. (d) Same as (c) averaged over four network representations, and error bars represent 1 standard error of the mean. Example shown for driving frequency of 21.5 Hz.

Inhibition can play additional, critical role within this framework. For the resonance frequency shift mechanism to be a general coding strategy, it has to be successfully applied to separate multiple competing neuronal representations. We show that separate heterogeneities can be selectively activated and dynamically switched through the use of targeted lateral inhibition (see methods). Excitatory neurons within competing heterogeneities mutually inhibit each other so that activation of one heterogeneity shifts the resonance of another heterogeneity away from the frequency of driving oscillation. Figure 7 shows an example network with two heterogeneities of identically enhanced synaptic coupling (σ = 4). In this case, the heterogeneities do not overlap and share basic excitatory connectivity (σ = 1) between each other and across the rest of the network. In addition, there are targeted inhibitory connections originating at one heterogeneity and targeting neurons in the other heterogeneity. Sub-threshold input applied to a subset of neurons within each heterogeneity selectively enhances the firing rate in that heterogeneity, but no enhanced firing occurs when input is applied to regions of the network outside of either heterogeneity (Fig. 7a,b). Thus, the heterogeneities can be selectively and dynamically activated. Similar strategy (targeted inhibition) could possibly be applied to multiple and overlapping representations.

The resonance shift mechanism not only selectively activates competing heterogeneities, but also dynamically controls switching the activation between heterogeneities. Activation of one heterogeneity blocks the activation of the other in a winner takes all fashion (Fig. 7c). We change the fraction of stimulated neurons (i.e. the neurons are driven with additional sub-threshold dc current) in heterogeneity 2, while the number of stimulated neurons in heterogeneity 1 remains constant (Fig. 7c,d). The heterogeneity 1 remains activated without significant change until the number of stimulated neurons in heterogeneity 2 exceeds that of heterogeneity 1. At that time there is a rapid switch in activation between the heterogeneities as heterogeneity 1 completely deactivates while heterogeneity 2 is fully activated. Thus, resonance shifts facilitated by targeted inhibitory coupling can separate network activity patterns and dynamically switch between activated network regions through sub-threshold input bias. Finally, in Figure 7d, we show that these results are robust against independent network realizations.

**Figure 8:**
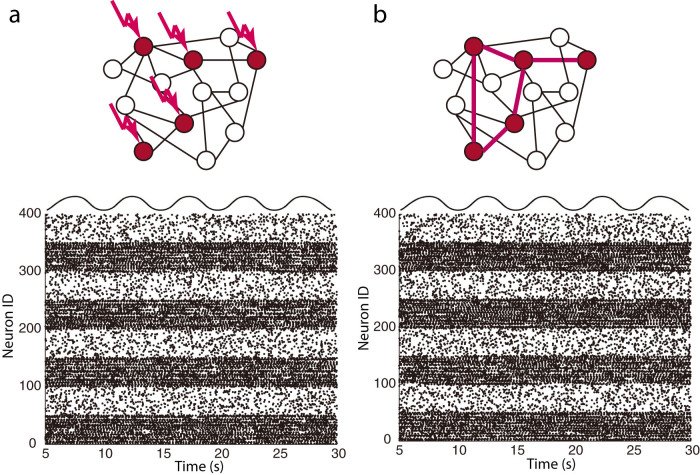
Resonance frequency shifts provide a mechanism for both functional and structural distributed network formation. (a) Sub-threshold input (arrows) driving a spatially distributed subset of the network (red circles), shifts the resonance frequency of those neurons creating a functional heterogeneity in the network, reflected in selective activation of functional clusters in the raster plot below. Learning mechanisms can strengthen these connections creating a distributed structural heterogeneity. (b) Distributed structural network heterogeneity (red circles) with additional coupling weight (thicker edges) is selectively activated based on the resonance frequency of sub-threshold input. Sub-threshold driving oscillations picture above raster plots are not to scale.

Finally, we show that the neuronal representation can also be distributed throughout the network and the resonance frequency shift can act as a feature binding mechanism for such a distributed representation. We illustrate how such a representation can be formed based on external input and easily retrieved based on intrinsic network dynamics (Fig. 8). Initially, for a network with no additional coupling (i.e. no stored memory), a small additional sub-threshold current bias is given to a population of neurons spatially distributed in the network. The source of this additional current could be activation from sensory input. This current shifts the resonance frequency of neurons receiving the additional current into resonance with the driving oscillation (Fig. 8a). The distributed population is activated, forming a functionally correlated ensemble. Learning rules can strengthen these connections creating a structural heterogeneity that can later be reactivated with the same resonance frequency shift mechanism when no biasing current is present. We heuristically show the effect by artificially strengthening the connectivity between the same neuronal populations (Fig. 8b).

## Discussion

It has long been assumed that input from our sensory environment affects neuronal activity, which alters the strength of the synaptic connections between neurons, forming an interactive feedback between network structure and its activity patterns. However, little is known about how large scale network activity results from modifications in neuronal coupling strength and network connectivity, or the role of oscillations in information processing. Here we provide a robust mechanism for spatial temporal pattern formation and separation based on the interaction of resonance phenomena in individual neurons, large scale oscillatory rhythms, and heterogeneities in network connectivity.

We demonstrated that regions of higher coupling can provide additional current to neurons within a network heterogeneity, shifting their resonance curves and leading to differential activation properties in their firing frequency and phase locking with dynamically changing sub-threshold input. In addition, the fast response times of the model networks to dynamically shifting resonances is similar to the timescales for enhanced firing frequencies observed in the brain during pattern identification tasks^29^,^30^,^31^.

These networks do not need to be limited to excitatory connections only; in fact the separation mechanism is enhanced by interactions with inhibitory neurons, which can allow for separation and switched between multiple representations. The described mechanism works well within known biological constraints of the brain^14^,^15^ and reproduces a number of experimental findings in terms of neuronal dynamics during memory retrieval^21^,^22^,^23^.

Until now two, to some extent competing, population coding strategies have been proposed: rate^33^ and spike-timing (synchrony) dependent^9^,^34^,^35^ coding mechanisms. Neuronal activity patterns indicative of both of these schemes have been observed. Rate-coding is generally easy to explain and intuitive to understand as greater supra-threshold input can provide higher frequency rates. While understanding of the spike-timing dependent mechanisms underlying the formation of content dependent, network wide, brief, and synchronous population bursts remains elusive. It is also unclear how rate coding can provide the content dependent, dynamic pattern separation^36^ needed for brain information processing.

The mechanism that we propose, based on sub-threshold input dependent resonance frequency shift, provides the theoretical solution to these inconsistencies and, furthermore, it links the two coding mechanisms together. Neurons that are in resonance with a given input oscillation will fire with higher frequency rates and also will phase lock to the oscillation, generating increased synchrony among themselves. Thus an input dependent resonance frequency shift provides means for dynamic content dependent grouping in frequency as well as phase domains.

While it clearly remains to be seen whether this mechanism plays a role in brain's information processing, in general it is not limited to biological neuronal networks but it provides a universal dynamical mechanism for information processing that can be applied to any network with oscillatory nodes.

Finally, it is worth noting that we used a linear dependence of resonant frequency on input. There are indications that this dependence could be non-linear due to type of transition from spiking to non-spiking neuronal state, specifically for type II neuronal excitability (subcritical Hopf bifurcation)^37^,^38^,^39^.

## Methods

### Network of Modified Resonate-and-Fire Neurons

The dynamics of each neuron is described by Resonate-and-Fire Neurons^24^ modified with a current dependent resonance frequency with the following equations: 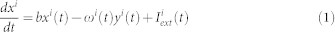

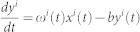
where 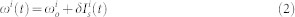
and 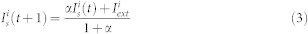


Here x^i^ is the internal current and y is the voltage of i^th^ neuron in the network and ω^i^ is the resonance frequency (or eigenfrequency) of the i^th^ neuron. It shifts from its intrinsic value ω_o_^i^ and is proportional to the current input I_s_^i^, with proportionality constant δ (Eq. (2)). I_s_^i^ is a slowly changing function (α = 10^5^) of the current input term I_ext_^i^ in Eq. (1), which in turn is defined as the sum of the synaptic coupling and sub-threshold oscillatory input current: 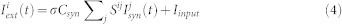
where 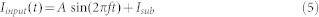


Here C_syn_ is the strength of synaptic coupling, σ is an additional weight multiplier to represent heterogeneities in network connectivity strength, S^ij^ is the adjacency matrix, and I_syn_^j^ is excitatory post-synaptic current input. We model I_syn_^j^ with a double exponential pulse that lasts approximately 15 ms. Eq. (5) describes the sub-threshold oscillatory input current, where A is the amplitude of oscillations, f is the driving frequency, and I_sub_ is sub-threshold external current. When the voltage reaches threshold, the current and voltage variables are reset to 0, and the neuron cannot receive input for a 5 ms refractory period. The neurons are additionally driven by noise; we set the voltage variable y^i^ above threshold with a given probability p_N_. For all simulations unless otherwise stated, the parameters are b = −1, ω_o_ = 100 (corresponds to ~16 Hz), δ = 40, σ = 4, C_syn_ = 5, A = 3, I_sub_ = 10, α = 10^5^. Eq. (1) is integrated using Euler's method, with a time step of h = 10^−5^. Four hundred neurons are connected into a 1D sparsely connected networks using the Small-World^25^ framework, with connectivity radius R = 3 and rewiring probability p = 0.1 unless otherwise stated.

### Modeling the Inhibitory Network

To study the potential impact of inhibitory neurons on the proposed pattern separation mechanism, we coupled an inhibitory network of 400 integrate-and-fire^32^ neurons to the excitatory resonate-and-fire networks. For simplicity we chose to use the integrate-and-fire model, where the dynamics of inhibitory neurons are given by: 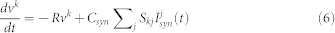


Where v^k^ is the voltage of the k^th^ inhibitory neuron, R is the membrane leak constant, C_syn_ is the strength of synaptic coupling, S_kj_ is the adjacency matrix between the k^th^ inhibitory neurons and the j^th^ other neurons in the network (where j spans both excitatory and inhibitory neurons) and I_syn_^j^ is the input current received. All parameters are the same as for the excitatory network with the following exceptions. C_syn_ is negative for inhibitory synapses. Network connectivity between inhibitory neurons is random, excitatory to inhibitory connections are local, and inhibitory to excitatory connections target randomly the whole network except the heterogeneity itself. To identify to what degree the excitation and inhibition are balanced in the network, we quantify the inhibitory network strength as a ratio of the average inhibitory synaptic input to average excitatory synaptic input that excitatory neurons receive integrated over a 3 second time window of the simulation.

### Targeted Inhibition

For the simulations on dynamic switching between competing heterogeneities, the effect of inhibition was modeled with direct, targeted inhibitory connectivity between the cells belonging to different heterogeneities. That is, all neurons within one heterogeneity had weak, targeted inhibitory connections, of strength C_syn_ = 2.5, to all cells within the second heterogeneity.

### Calculation of Phase and Mean Phase Coherence

We created continuous signals of the network activity within and outside of the heterogeneity, by collapsing all spike trains within given region into one aggregate spike train and convolving each signal with a Gaussian function of 10 millisecond width. To quantify the phase locking we computed the instantaneous phases of the network activity and the sub-threshold oscillatory input using the equation: 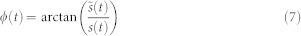
where the instantaneous phase *φ*(t) for each signal is found using the Hilbert transform 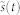 of the signal s(t) defined as: 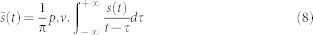


The p.v. denotes the Cauchy principal value. The Mean Phase Coherence (MPC) is given by: 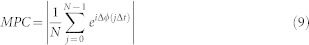
where Δφ is the phase difference between the instantaneous phase of the network activity signal and the instantaneous phase of the sub-threshold oscillatory input current, N is the number of timesteps in the simulation, and Δt is the step size.

### Calculation of Firing Rate Response Time

Neurons were driven with a subthreshold oscillatory input with constant frequency and given an additional subthreshold current step 10 seconds into the simulation to shift their resonance. To quantify the onset of firing, we calculated the average firing rate over a moving time window of 500 ms. Similar results were observed for 250 and 750 ms time-windows.

## Author Contributions

E.S. and M.Z. proposed the theoretical approach and wrote the manuscript. E.S. performed the computer simulations. Both authors reviewed the manuscript.
